# Linezolid Pharmacokinetics in Critically Ill Patients: Continuous Versus Intermittent Infusion

**DOI:** 10.3390/antibiotics13100961

**Published:** 2024-10-11

**Authors:** Ligia-Ancuța Hui, Constantin Bodolea, Adina Popa, Ana-Maria Vlase, Elisabeta Ioana Hirișcău, Laurian Vlase

**Affiliations:** 1 Pharmaceutical Technology and Biopharmacy Department, Faculty of Pharmacy, University of Medicine and Pharmacy “Iuliu Hatieganu”, 400012 Cluj-Napoca, Romania; ligia.secara@umfcluj.ro (L.-A.H.); laurian.vlase@umfcluj.ro (L.V.); 2 ICU Department, University Clinical Municipal Hospital, 400139 Cluj-Napoca, Romania; constantin.bodolea@umfcluj.ro (C.B.); ihiriscau@umfcluj.ro (E.I.H.); 3 ICU Department, Faculty of Medicine, University of Medicine and Pharmacy “Iuliu Hatieganu”, 400006 Cluj-Napoca, Romania; 4 Clinical Pharmacy Department, Faculty of Pharmacy, University of Medicine and Pharmacy “Iuliu Hatieganu”, 400012 Cluj-Napoca, Romania; 5 Pharmaceutical Botany Department, Faculty of Pharmacy, University of Medicine and Pharmacy “Iuliu Hatieganu”, 400012 Cluj-Napoca, Romania; gheldiu.ana@umfcluj.ro; 6 Nursing Department, Faculty of Medicine, University of Medicine and Pharmacy “Iuliu Hatieganu”, 400089 Cluj-Napoca, Romania

**Keywords:** continuous infusion, critically ill patient, ICU, intermittent infusion, linezolid, loading dose

## Abstract

Background: Linezolid has been found to have considerable interindividual variability, especially in critically ill patients, which can lead to suboptimal plasma concentration. To overcome this shortcoming, several solutions have been proposed. These include using loading dose, higher maintenance doses, and dose stratification according to the patient’s particularities, therapeutic drug monitoring, and drug administration via continuous infusion (CI) instead of intermittent infusion (II). In the present study, we aim to compare the pharmacokinetic (PK) parameters of linezolid after administration as II versus CI to critically ill patients. Methods: In a prospective study conducted in an intensive care unit, we compared the same two daily doses of linezolid administered via II versus CI. The serum concentration was measured, and pharmacokinetic parameters were calculated. The pharmacokinetic/pharmacodynamic (PK/PD) indices for efficacy chosen were area under the concentration–time curve at steady state divided by the minimum inhibitory concentration over 80 (AUC24–48/MIC > 80). Results: Greater serum concentration variability was observed in the II group than in the CI group. The %T > MIC > 80% was achieved for MICs of 1 and 2 µg/mL 100% of the time, whereas for the II group, this was 93% and 73%, respectively. AUC24–48/MIC > 80 was reached in 100% of cases in the CI group compared with 87% in the II group for a MIC of 1 µg/mL. Conclusions: The two infusion methods may be used comparably, but utilizing CI as an alternative to II may have potential benefits, including avoiding periods of suboptimal concentrations, which may enhance safety profiles and clinical outcomes. Considering the relatively few studies performed on linezolid to date, which are increasing in number, the results of the present study may be of interest.

## 1. Introduction

Linezolid is an oxazolidinone antibiotic that was first approved by the Food and Drug Administration in 2000. Its antibacterial activity is due to the inhibition of protein production by binding to the 50 S ribosomal subunit of the bacteria [1]. The approved indications for linezolid include community- and hospital-acquired pneumonia, as well as skin and soft tissue infections caused by Gram-positive bacteria, such as *Staphylococcus aureus*; methicillin-sensitive (MSSA) and methicillin-resistant (MRSA) strains; or *Streptococcus* spp., including multidrug-resistant strains. It is also indicated for vancomycin-susceptible or vancomycin-resistant *Enterococcus faecium* (VREF) infections with concomitant bacteremia. Furthermore, it has been used off-label in other Gram-positive bacterial infections, like central nervous system (CNS) infection, intracranial and spinal abscess, cystic fibrosis exacerbations, endocarditis, meningitis, osteomyelitis, discitis, prosthetic joint infection, septic arthritis, toxic shock syndrome, and tuberculosis [2]. It has no activity against Gram-negative bacteria and can be administered parenterally or orally, with no requirement for dose adjustment when switching from one route to the other, and without the need for renal or hepatic adjustment [3,4,5].

Linezolid is a time-dependent antibiotic, with its action best characterized by the following pharmacokinetic/pharmacodynamic (PK/PD) indices: area under the concentration–time curve at steady state divided by the minimum inhibitory concentration (AUC/MIC) typically of 80–85 and the percentage of time that the plasma concentrations surpass the MIC (T > MIC) in a proportion of 85–100% [6,7]. Concurrently, an optimal clinical response was correlated with steady-state concentrations (Css) within the range of 2–10 µg/mL. Nevertheless, a 50% increase in the incidence of thrombocytopenia was observed when Css exceeded 10 µg/mL [8].

It has been stated that linezolid exhibits considerable inter-individual variability and that the standard dose may not be optimal for all individuals [9]. In critically ill patients, it was observed that achieving the optimal concentration for a clinical response was not feasible through traditional intermittent infusion (II) of 600 mg every 12 h via a 1–2 h infusion, which is the approved dose and infusion duration [10]. Some patients may benefit from alternative dosage regimens that are off-label for example the use of a loading dose, continuous infusion (CI) or/and higher doses of linezolid to achieve the PK/PD indices. Such patients may be those who are obese, those who suffer from acute respiratory distress syndrome, and those who have augmented renal clearance (ARC), as well as those who are treated for infections with bacteria that have a MIC of at least 2 µg/mL [4].

To the best of our knowledge, there have been 14 articles published to date discussing the CI of linezolid, considering approximately 400 patients in total, with half of them included in studies published over the past two years. Therefore, the interest in CI has grown, and scientists are becoming more confident in this type of administration. In terms of comparative studies between linezolid administered as CI vs. II, there are only eight studies, with less than 100 patients in total. Furthermore, only two of these studies included more than 15 patients per group, with some of them comprising a sole case or series of cases. It should be noted that not all studies were conducted on critically ill patients and used a loading dose before initiating the CI of linezolid. Critically ill patient studies are inherently challenging to perform, as these patients may exhibit several pathophysiological alterations and require specialized medical devices to support vital organ functions. Additionally, they often require rapid and continuous monitoring and treatment change, which presents a significant challenge in clinical settings (Supplementary Data, Table S1) [10,11,12,13,14,15,16,17,18,19,20,21,22,23,24,25,26,27,28].

The results of current research studies comparing CI with classical II of linezolid in critically ill patients have yielded encouraging conclusions, suggesting that continuous infusion may offer a greater likelihood of achieving clinical cure, as well as greater success in attaining PK/PD indices and a reduced risk of adverse reactions [16,18,24].

In the present study, we aim to compare the PK parameters of linezolid after administration as II versus CI to critically ill patients from the intensive care unit.

## 2. Results

### 2.1. Study Population Characteristics

A total of 55 patients received linezolid during the study period; however, 21 were excluded from this study due to failure to meet the inclusion criteria. Three patients from the II group and one from the CI group survived for less than 24 h after the start of the linezolid infusion, and one patient had a sampling error, and they were lost from the study. Thus, a total of 29 patients were included in this study.

**Figure 1:**
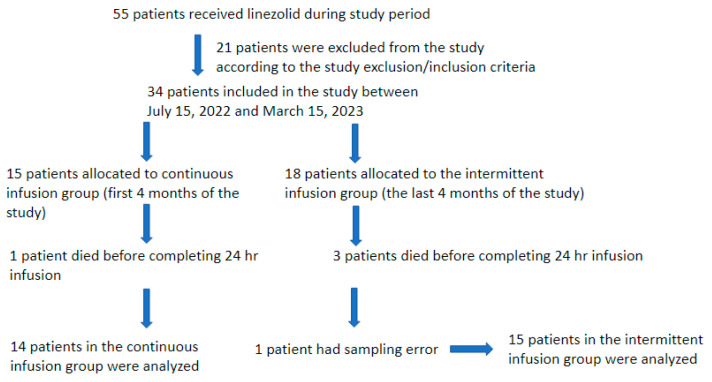
Patients’ selection flowchart.

The initial 14 patients included in the study were allocated to the CI group, and the subsequent 15 patients were allocated to the II group (Figure 1). Table 1 presents a comprehensive summary of all patients’ demographic, anthropometric, and prescription data before linezolid treatment initiation. The study population comprised 7 women and 22 men, all of whom were Caucasian. The age range of the participants was between 45 and 87 years, and their body mass index (BMI) ranged from 15.7 to 41.7 kg/m^2^. The observed differences between the two groups were not statistically significant, except for age. In the II group, patients were observed to be older (median age of 75 years old vs. 72 years old in the CI group; *p* = 0.046). The 30-day survival rate in the CI group was 50% (7 out of 14 patients), while in the II group, it was 66.66% (10 out of 15). One patient in the CI group and three patients in the II group died during linezolid therapy or in the first 48 h following its discontinuation. For the patients who died during the 30-day follow-up period, the average number of days of survival was 16 in the CI group and 8 days in the II group. Their cause of death was not linezolid’s failure to clear Gram-positive bacteria.

Eight patients from the CI group and seven from the II group started linezolid therapy on the first day of their admission to the ICU. Notably, 19 patients (10 from the CI group and 9 from the II group) were admitted to the ICU directly from the emergency department, while 10 patients (4 from the CI group and 6 from the II group) were transferred from another medical or surgical unit having a greater risk of hospital-acquired infection.

The patients included in the study with creatinine clearance (CrCl) above 130 mL/min were three in the CI group and none in the II group; patients with CrCl between 61 and 130 mL/min were two in the CI group and six in the II group; patients with CrCl between 31 and 60 mL/min were four in the CI group and six in II group; patients with CrCl between 15 and 30 mL/min were three in the CI group and two in the II group; and patients with CrCl 0 and 14 mL/min were two in the CI group and one in the II group. Patients included in the study with no hepatic impartment or mild hepatic impartment (Child–Pugh score A) were six in the CI group and eight in the II group, and patients with moderate hepatic impartment (Child–Pugh score B) were eight in the CI group and seven in the II group. Patients with Child–Pugh score C were not included in this study.

We did not find statistically significant differences between groups regarding the procalcitonin level. The median C-reactive protein was observed to be higher in the CI group, though this did not reach statistical significance (*p* = 0.08). 

Of the total of 29 patients included in this study, empirical therapy was initiated in 19 cases, while in the remaining 10 cases, a Gram-positive culture collected at admission prior to the commencement of therapy was validated. Regardless of whether empirical or targeted therapy was initiated, in the CI group, five *Enterococcus* and one *Staphylococcus* strains were identified, while in the II group, four *Enterococcus* and five *Staphylococcus* strains were identified. These results were obtained without consideration of the screening strains from axillar/rectal/nasal sites.

The diagnosis for linezolid treatment was septic shock; bacteriemia; or urinary, cutaneous, pulmonary, or abdominal infection. No statistical significance was found between the two groups in terms of the type of infection. 

The mean duration of linezolid treatment was 7.2 days (SD 3.1 days) in the CI group and ranged from 2 to 13 days, while in the II group, the mean was 5.8 days (SD 2.8 days), with a minimum of 2 days and a maximum of 10 days.

### 2.2. Pharmacokinetics (PK)

**Figure 2:**
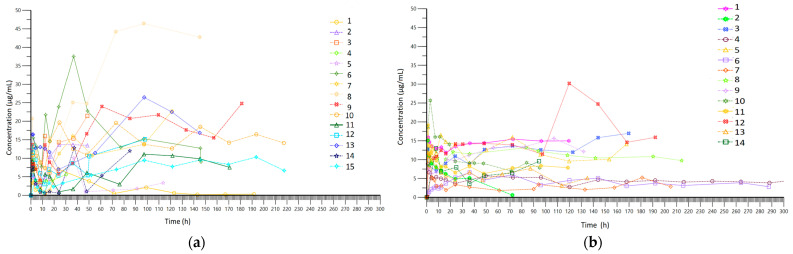
(**a**) Real sparse data (spaghetti plot) for time–concentration curve in the intermittent infusion (II) group; (**b**) real sparse data (spaghetti plot) for time–concentration curve in the continuous infusion (CI) group.

**Figure 3:**
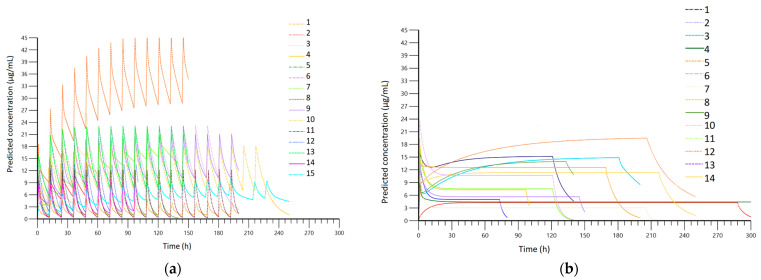
(**a**) Fitted serum concentration obtained after PK modeling in the intermittent infusion (II) group; (**b**) fitted serum concentration obtained after PK modeling in the continuous infusion (CI) group.

A total of 396 blood samples were analyzed. Figure 2 presents the concentrations observed in each group, while Figure 3 shows the results of pharmacokinetic modeling. The PK profiles from the CI group after modulation demonstrate linearity in terms of concentration, with no significant variations observed during treatment, except when the loading dose was administered. Values between patients ranged mainly from 3 µg/mL to 15 µg/mL. In the II group, values ranged mainly from 0 µg/mL to 25 µg/mL, with more variability during treatment. In Table 2 we have included the pharmacokinetic parameters obtained in each group.

**Figure 4:**
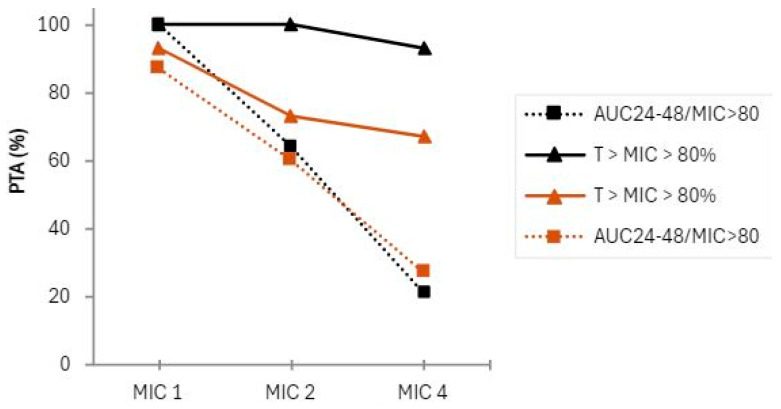
Probability of target attainment (PTA) in terms of PK/PD indices: black line, full and dotted: the CI group; brown line, full and dotted: ed: the II group.

The established PTA was AUC_24–48_/MIC > 80, and T > MIC > 80% at the MICs of 1, 2, and 4 µg/mL. Table 3 illustrates that, for the CI group, the T > MIC > 80% was achieved for MICs of 1 and 2 µg/mL 100% of the time, and it was achieved 93% of the time at a MIC of 4 µg/mL, whereas for the II group, this parameter was achieved 93%, 73%, and 67% of the time in MICs of 1, 2 and 4, respectively. Regarding AUC_24–48_/MIC > 80, the PTA was reached in 100% of cases in the CI group compared with 87% in the II group for a MIC of 1 µg/mL. For the same parameter, the PTA was reached in 64% of cases in the CI group and in 60% of cases in the II group for a MIC of 2 µg/mL. At a MIC of 4 µg/mL, the PTA was even lower (Figure 4).

In Table 4, different covariates that could influence the serum concentrations of linezolid regardless of the infusion method are listed. Statistically significant differences were noted in several parameters. For example, gender significantly influenced the distribution clearance (*p* = 0.018), the distribution constant from the central to the peripheral compartment (*p* = 0.025), the maximum serum concentration (*p* = 0.024), and the distribution volume of the peripheral compartment (*p* = 0.023). In addition, age had an impact on the distribution constant from the central hydrophilic compartment to the peripheral lipophilic one, but it was not statistically significant (*p* = 0.097), and it also influenced the distribution clearance, but it did not pass the threshold of statistical significance (*p* = 0.084). No statistically significant changes in the analyzed pharmacokinetic parameters due to the albumin level in the body were observed in the analyzed group. The extracorporeal purification methods, such as continuous venovenous hemodiafiltration (CVVHDF) or intermittent dialysis (HD) 3 times per week, did not show a statistically significant influence. The liver and kidney function had an impact on the elimination constant with *p*-values of 0.054 (very close to statistically significant) and 0.095, respectively. The liver function was found to have an impact even when we excluded patients with liver failure (those in the Child–Pugh C category) from the study.

## 3. Discussion

Even if there is variability between the two patient groups with respect to age, the manufacturer’s specifications include the same recommended dose of 1200 mg/day, irrespective of the patient’s age [3], and this aligns with the dose that we used in our study.

We obtained higher mean Vss than other authors obtained in healthy volunteers [29,30] probably due to our patients’ particularities like old age, critically ill, with resuscitation fluid treatment, and ongoing CVVHDF. Cmax, in healthy volunteers, was 15 µg/mL (SD 3 µg/mL), and in critically ill, it was 16.7 µg/mL (SD 3.11 µg/mL) when using intermittent infusion and 8.96 µg/mL (SD 0.96 µg/mL) when using continuous infusion [14]. Adembri et al. obtained a Cmax of 11.5 µg/mL in the CI group and 13.1 µg/mL in the II group in critically ill ICU patients [28]. In our study, Cmax was 10.91 µg/mL (SD 3.82 µg/mL) in the II group and 13.58 µg/mL (SD 5.47 µg/mL) in the CI group. Our data are consistent with the findings of Adembri et al., from the Cmax point of view. We obtained a similar AUC_0–24_ to that reported by Boselli et al. in patients with intermittent infusion [26].

Dong et al. found a T > MIC between 53.4% and 100% at MIC = 2 µg/mL in intermittent infusion [31]. In our study, the same parameter was between 31.7% and 100% in the II group, but in the CI group, it was 100% for all patients. These results make the CI superior to II with a greater chance of clinical cure.

Ide et al. performed simulations using intermittent infusion at specific renal functions, and they obtained different PTA values for achieving an AUC/MIC  ≥  80 for specific MICs. In patients with normal renal function, PTA was low at MIC  =  4 µg/mL, in line with our data. PTA was 77.2% for MIC  =  2 µg/mL using a standard dosing regimen (600 mg every 12 h) [32]. Using the same PTA, without renal function stratification, we obtained 60% in the II group and 64% in the CI group. This lower percentage may be a consequence of not having renal function stratification in our study.

The coefficient of variation was higher in the II group than in the CI group. However, in most cases within the CI group, the coefficient of variation still surpassed 20%. This is attributable to the loading dose, which quickly elevates serum concentrations before stabilizing at a steady state with minimal fluctuations.

Bandín-Vilar et al. stated that the target Cmin should be between 2 and 7 µg/mL, and Cmin higher than 7.5–22.1 µg/mL is correlated with a higher risk of toxicity [9]. Considering that blood samples were collected at points during the course of therapy, their graphical representation (Figure 2a,b) is difficult to interpret; therefore, it is necessary to perform pharmacokinetic modeling. A comparison of the PK modeling profiles of all patients in the II group (Figure 3a) reveals a high degree of intra-individual variability, with concentrations ranging from below the therapeutic concentration (0 µg/mL) to above the therapeutic concentration (25 µg/mL) with considerable frequency. In contrast, the concentration profile obtained in the CI group demonstrated a consistent range of values between 3 µg/mL and 15 µg/mL with less intra-individual variation (Figure 3b). Therefore, the risks associated with therapy, including therapeutic failure and adverse reactions, are expected to be lower in the CI group.

Considering our data analysis, and the results of other studies [18,28], it can be concluded that the efficacy of linezolid administered via CI is expected to outperform that of II, with a corresponding reduction in the likelihood of adverse events.

Additionally, the influence of various covariates on the serum concentrations of linezolid, irrespective of the infusion method, was investigated. At the level of some covariates, statistically significant differences were observed, as expected. For example, gender influenced the distribution clearance and the maximum serum concentration, probably due to the different ratios of muscle tissue versus adipose tissue between the sexes. This suggests that the sizes of the central and peripheral compartments differ between females and males. Additionally, when considering age, women generally have a higher proportion of adipose tissue compared to men, which causes alterations in the water compartment proportion. For the same reasons, the distribution constant from the central to the peripheral compartment and the volume of the distribution of the lipophilic peripheral compartment showed statistically significant differences between the sexes. Furthermore, probably because of the same reasons, age was found to impact the distribution clearance and the distribution constant from the central to the peripheral compartment but probably because of the small group size, it did not pass the threshold of statistical significance. The hepatic and renal function was found to influence the elimination constant in a non-statistically significant manner. Further larger studies are needed to confirm whether liver and kidney function do in fact influence linezolid elimination.

Given the small number of patients included in this study, the data regarding the impact of renal function on linezolid blood concentration cannot be generalized, but we consider two patients from the continuous infusion group as an example: one with augmented renal function (ARC patient) and one with severe renal failure, with CrCl below 15 mL/min (SRF patient) but not on chronic dialysis. AUC24–48 was 124 h*µg/mL for the ARC patient and 261 h*µg/mL for the SRF patient. This AUC difference was also confirmed by the difference in Css, which, at 48 h after the initiation of continuous infusion, was 5.03 µg/mL for the ARC patient versus 11.97 µg/mL for the SRF patient. The half-life was 4.83 h for the ARC patient versus 27.99 h for the SRF patient.

Even if the extracorporeal purification methods (CVVHDF and HD) may determine a greater volume of distribution, no statistically significant alterations of the pharmacokinetic parameters were identified in the analyzed group, which may be a consequence of the limited number of participants in this study.

This is the third largest comparative study of linezolid administered as a continuous infusion versus intermittent infusion to date, as shown in Supplementary Data, Table S1 [16,17]. Barrasa et al. stratified patients according to CrCl, obtaining Css of 7.9 µg/mL in patients with continuous infusion and CrCl of 40–130 mL/min and 2.8 µg/mL in those with ARC. The differences were also maintained in the case of intermittent infusion, between the two categories of patients in terms of Cmax and Cmin [17]. These data are in line with those of our examples mentioned above. Warda et al., in the largest comparative study of the two proposed modes of administration, do not provide pharmacokinetic parameters, as their investigation was a safety and efficacy study of linezolid. This study concludes that continuous infusion is superior to intermittent infusion in terms of clinical response and presents a lower risk of adverse reactions (thrombocytopenia) [16].

While we recognize the limitations of our study, including the small sample size and variability in age distribution between groups, it is important to emphasize that the findings from this pilot study provide valuable insights into the pharmacokinetics of linezolid in critically ill patients. The study highlights potential differences in drug exposure between continuous and intermittent infusion, which could have meaningful implications for optimizing therapeutic strategies and improving clinical outcomes in this vulnerable patient population.

Given the small sample size and its effect on the statistical power of our results, we agree that additional studies with larger patient cohorts are essential. A more comprehensive population study would allow for a deeper understanding of how different patient factors influence drug exposure and efficacy. Such studies would ultimately help to refine dosing guidelines and enhance the clinical management of linezolid therapy, ensuring safer and more effective treatment.

## 4. Materials and Methods

### 4.1. Setting

This was a prospective, open-label, single-center, two-arm study, conducted in accordance with the ethical standards outlined in the 1964 Declaration of Helsinki and approved by the local Ethics Committee. It was registered with ClinicalTrials.gov under the unique identifier no. NCT05801484. This study was carried out between July 2022 and March 2023 at the intensive care unit of the University Clinical Municipal Hospital in Cluj-Napoca. Written informed consent was obtained from the participants or a family member.

### 4.2. Study Population

The inclusion criteria were as follows: female or male critically ill patients who were admitted to the intensive care unit, over the age of 18, with a Gram-positive suspected or documented infection for which intravenous linezolid was prescribed by the attending physician. We considered a critically ill patient as a patient admitted to the intensive care unit (ICU), with a Sequential Organ Failure Assessment Score (SOFA) ≥ 2 and with multiple comorbidities [33]. The exclusion criteria were a severe hepatic failure, classified as Child–Pugh C, and refusal to sign informed consent. The survival expectancy was not an exclusion criterion, but we excluded patients who did not survive at least 24 h after linezolid initiation from the study.

The patients who were diagnosed with septic shock or other severe infections with high suspicion of resistant Gram-positive infection were admitted to the ICU via the emergency room or from medical/surgical units. A convenience allocation was employed, with patients enrolled in the first four months of the study being assigned to the CI group and those enrolled in the following four months to the II group. Given that the ward’s standard practice was to utilize CI, it was decided that the initial round of allocation should be allocated to the CI group.

The patient’s renal function was divided into five categories: augmented creatinine clearance at CrCl above 130 mL/min, CrCl between 61 and 130 mL/min, CrCl between 31 and 60 mL/min, CrCl between 15 and 30 mL/min, and CrCl 0 and 14 mL/min. From the hepatic function point of view, the patients were divided into two groups: with no hepatic impartment or mild hepatic impartment (Child–Pugh score A), and with moderate hepatic impartment (Child–Pugh score B). Patients with Child–Pugh score C were not included in the study.

### 4.3. Linezolid Therapy Initiation

The decision to initiate linezolid therapy was made not by the investigators but by the attending physicians according to their judgment regarding infection assessment and antibiotic initiation as the attending physicians did not participate in this study. Then, the investigators introduced the patients according to the inclusion/exclusion criteria in the study, in one of the two arms. 

After taking cultures, the patients were given linezolid (Fresenius Kabi AG, Homburg, Germany) along with another broad-spectrum antibiotic to cover Gram-negative bacteria (amoxicillin + clavulanate (Antibiotice SA, Iasi, Romania)/ampicillin + sulbactam (Antibiotice SA, Iasi, Romania)/ceftriaxone (Antibiotice SA, Iasi, Romania)/piperacillin + tazobactam (Fresenius Kabi AG, Homburg, Germany)/meropenem (Antibiotice SA, Iasi, Romania)/ceftazidime + avibactam (Pfizer, New York City, NY, USA).

### 4.4. Study Protocol

The CI group received a one-hour intravenous infusion of 600 mg (with a rate of 600 mg/h of linezolid as a loading dose followed by 1200 mg/day by continuous infusion (with a rate of 50 mg/h), as Boselli at al first proposed [26]. The II group received the approved dose regimen of 600 mg of linezolid infused in 1 or 2 h every 12 h with a rate of 300–600 mg/h [3]. The treatment duration was between 2 days and 13 days based on the physician’s decision.

### 4.5. Blood Samples

**Figure 5:**
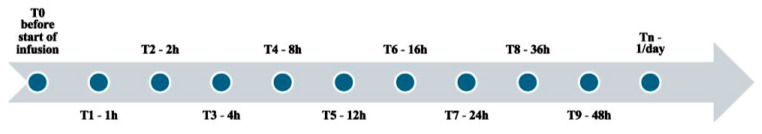
Sampling regimen: T0: first blood sample drawn before the start of infusion; T1–T9: blood samples drawn at a specific number of hours from the start of infusion; Tn: blood samples drawn one/day after the first 48 h of infusion.

Nurses not involved in the study were appointed to administer the antibiotic and to obtain blood samples for each patient. The blood samples were obtained at regular intervals from each volunteer. These commenced immediately prior to the initiation of infusion (T0) and then again at 1, 2, 4, 8, 12, 16, 24, 36, and 48 h from the start of the drug infusion. Subsequently, a single daily sample was taken until the completion of the treatment (Figure 5). Two milliliters of blood were collected for each sample in a vacutainer containing a clot activator (Beckton Dickinson, Franklin Lakes NJ, USA)and stored at 2–8 °C, away from light for a maximum of 24 h. Subsequently, the investigator assigned a code to each sample without indicating its group origin, centrifuged (Rotafix 32A, Hettich, Tuttlingen, Germany) it at 3500 rotations per minute for 10 min, and removed the serum, and then the sample was stored at −80 °C until analysis. Once all samples from both groups had been collected, they were transported to another facility for concentration determination.

### 4.6. Variables/Patient Parameters 

Sociodemographic data were collected on age and gender, origin, height, and weight, with the calculation of body mass index (BMI = weight/height^2^). Additionally, data on patients’ comorbidities were extracted from their medical records. To quantify the comorbidities, the Charlson Comorbidity Index (CCI) was employed, which is a clinically useful tool for validating the patient’s condition and an independent predictor of long-term survival [34].

A number of blood parameters were determined as part of the standard care procedure, including the number of white blood cells, the number of platelets, and the number of neutrophils and lymphocytes. These were recorded at the time of linezolid therapy initiation. Additionally, neutrophil-to-lymphocyte ratios (NLRs) and platelet-to-lymphocyte ratios (PLRs) were calculated. Biological or pathological covariates (i.e., renal and hepatic function and renal replacement therapy) were collected in order to identify their effect on serum concentrations of linezolid. The Simplified Acute Physiology Score II (SAPS II) and Sequential Organ Failure Assessment Score (SOFA) were calculated for each patient at the time of admission in order to assess the mortality risk. In addition, data pertaining to infection, including the type of infection and the results of microbiological cultures for Gram-positive infection confirmation, were recorded. 

### 4.7. Bioanalytic Assessment

An LC-MS method, specifically validated for serum analysis prior to the clinical study, was used to determine the concentration of linezolid, ensuring both the accuracy and precision of the drug concentration measurements in this matrix.

The HPLC system (1100 series, Agilent Technologies, Santa Clara, CA, USA) was coupled to an Agilent MSD SL Ion Trap detector (Agilent Technologies, Santa Clara, CA, USA) equipped with an electrospray ionization (ESI) interface, operating in the positive ionization mode. Chromatographic and mass spectrometric data acquisition were conducted using Chemstation software from Agilent, Santa Clara, CA, USA, version B.01.03, and LC-MSD Trap Control (Bruker Daltonik, GmbH, Bremen, Germany), version 5.3. Data processing was completed with LC-MSD Quant Analysis software (Bruker Daltonik, GmbH, Bremen, Germany), version 1.7.

Separation was achieved using a Kinetex Core–Shell C18 reversed-phase analytical column (Phenomenex, Torrance, CA, USA) (50 × 2.1 mm, 2.6 µm particles) with a mobile phase consisting of 1 mM ammonium formate (Sigma Aldrich Chemie GmbH, Schnelldorf, Germany and acetonitrile (Merck KGaA, Darmstadt, Germany) (75:25, *v*/*v*) at a flow rate of 0.4 mL/min. The column was maintained at 40 °C using a G1316 A oven (Agilent Technologies, Santa Clara, CA, USA). All solvents were filtered through 0.45 µm Minisart^®^ RC25 syringe filter (Sartorius Lab Instruments, Göttingen, Germany (Sartorius Lab Instruments, Göttingen, Germany) and degassed in an ultrasonic bath (Sonorex Super RK 100 H, Bandelin Electronic GmbH & Co. KG, Berlin, Germany). The injection volume was 1 µL.

For mass spectrometric analysis, nitrogen was the drying gas (10 L/min at 350 °C) and nebulizing gas (45 psi). The capillary voltage was set to 3000 V, with the capillary exit voltage (fragmentor) at 88 V and the trap drive at 34 V. The precursor ion (*m/z* 338) was selected with an isolation window of 3 *m/z* and fragmented at a 0.75 V amplitude, with a cut-off at 98 *m/z*. The scan range was 293–299 *m/z*, and the monitored ion transitions using a mass spectrometer (Agilent Technologies, Santa Clara, CA, USA) was from *m/z* 338.0 to 296.2.

The analytical standard for linezolid was supplied by SynFine Research, Ontario, Canada. Calibration was conducted over a concentration range of 0.256 to 20.130 µg/mL using quadratic least-square regression, with an R^2^ value greater than 0.995. The limit of quantification (LOQ) for linezolid was determined to be 0.256 µg/mL.

Sample pretreatment involved vortex-mixing of 0.2 mL of serum with 1 mL of acetonitrile in a 1.5 mL Eppendorf tube for 10 s, followed by centrifugation at 12,000 rpm for 3 min using Centrisart^®^ D-16C centrifuge (Sartorius Lab Instruments, Göttingen, Germany). A 0.1 mL aliquot of the supernatant was transferred into a fresh tube, mixed with 0.9 mL of 1 mM ammonium formate, and shortly vortexed. Finally, 200 µL of the resulting solution was transferred to an HPLC autosampler vial (Agilent Technologies, Santa Clara, CA, USA), and 1 µL was injected into the column [35].

### 4.8. Data Analysis

Continuous variables are expressed as the mean or median and standard deviation and categorical variables as the absolute value with their percentage. We used the chi-squared test to compare the categorical variables. We used the Pearson correlation for linear correlations between continuous variables and the Spearman rank test for other variables. Statistical significance was set at a *p*-value ≤ 0.05. Data analyses were conducted using IBM SPSS Statistics for Windows, version 22.0 (IBM Corp., Armonk, NY, USA). 

The pharmacokinetic modeling employed in our study utilized both mono- and bi-compartmental models available in Phoenix WinNonlin, version 6.3 (Certara, Princeton, NJ, USA), which are well-established, standard models provided by the software. Model selection and discrimination were carried out by evaluating the Akaike Information Criterion (AIC), with the model showing the lowest AIC value being considered the best fit for the data. In our case, the bi-compartmental model demonstrated the best fit and was therefore selected for the final analysis. Several pharmacokinetic parameters were obtained and evaluated, such as the minimum, maximum, and steady-state serum concentration (Cmin, Cmax, and Css); the area under the curve (AUC); distribution macro-constants; (Alpha and Beta); half-life of the distribution phase (Alpha_HL); half-life of the given drug (Beta_HL); clearance (CL); distribution clearance (CLD2); elimination constant (K10); distribution constant from central (1) to peripheric (2) compartment (K12); distribution constant from peripheric (2) to central (1) compartment (K21); distribution volume of central compartment (V1) and peripheric compartment (V2); and the distribution volume at steady state (Vss).

The statistical analysis was performed using the descriptive statistics for the PK parameter module in Phoenix software. For the evaluation of the statistical significance of the influence of cofactors on PK parameters, we employed ANOVA through the linear mixed-effect module in Phoenix.

AUC_24–48_/MIC > 80 at MIC of 1, 2, and 4 µg/mL, and T > MIC > 80% were chosen as PK/PD indices to determine the PTA. Using the standards from the Clinical and Laboratory Standards Institute (CLSI), we considered the MIC breakpoint of ≤2 µg/mL for *Streptococcus* spp. and *Enterococcus* spp. and MIC ≤4 µg/mL for *Staphylococcus* spp. [36]. Recent studies have indicated that isolates tend to exhibit a distribution of approximately 50% to 50% between MIC levels of 1 µg/mL and 2 µg/mL [14,37]. Given the absence of MIC determination for our study, we proceeded as suggested in the literature, calculating the PK/PD indices using MICs of 1, 2, and 4 µg/mL [38].

## 5. Conclusions

This is the third largest comparative study of linezolid administered as a continuous infusion versus intermittent infusion to date, as we have previously shown.

The objective of this study was to ascertain the differences in pharmacokinetics between continuous and intermittent administration of the same daily dose of linezolid. The pharmacokinetic data obtained indicate that there were no differences between the two groups. Both groups exhibited similar peak concentrations and areas under the curve, with the CI group demonstrating less concentration variation throughout the course of treatment. It can therefore be concluded that the two infusion methods may be used in a comparable manner, but the potential benefits of utilizing continuous infusion as an alternative to intermittent infusion include the avoidance of periods of sub-therapeutic and supra-therapeutic concentrations, which may enhance safety profiles and clinical outcomes.

Continuous infusion provides a greater opportunity to attain the PK/PD indices at MICs of 1 µg/mL and 2 µg/mL, although for a MIC of 4 µg/mL, higher doses may be required.

The increasing microbial resistance to antibiotics and the rising prevalence of resistant Gram-positive bacteria, which present a significant financial burden and a public health issue [39,40], highlight the need for alternative strategies in the prevention of antibiotic resistance. The continuous infusion of linezolid may represent a cost-effective approach for this purpose. The association between continuous infusion and therapeutic drug monitoring may prove to be the optimal combination for the successful therapeutic use of linezolid in critically ill patients with pathological alterations. 

In the context of the currently approved intermittent infusion of linezolid, and the fact that there are an increasing number of studies prospecting continuous administration of antibiotics dependent on the time during which the concentration exceeds the minimum inhibitory concentration, the evaluation of linezolid administration by continuous infusion is of interest. Considering the relatively small number of studies performed for linezolid to date, the results of the present study may be of interest.

Given the study limitations, there is a need for populational studies to be able to generalize the results to an entire population.

## Figures and Tables

**Table 1:** Demographic, anthropometric, blood parameters/markers, and illness severity data of the study patients.

Variables			All Sample (*n* = 29) *n*, Mean or Median	II Group (*n* = 15) *n*, Mean or Median	CI Group (*n* = 14) *n*, Mean or Median	*p*-Value
Demographics	Female/Male		7/22	5/10	2/12	0.246
	Age (years)		75.0 (68.5–80.5)	75.0 (75.0–81.0)	72.0 (61.0–80.0)	0.043 *
	Weight (kg)		81.9 (19.0)	85.8 (14.5)	77.7 (22.7)	0.259
	Height (cm)		170.0 (170.0–175.0)	170.0 (165.0–175.0)	175.0 (170.0–176.25)	0.138
	BMI (kg/m^2^)		27.6 (6.00)	29.5 (5.2)	25.6 (6.4)	0.084
Blood parameters (at admission)	Leucocytes		13.4 (10.7–21.0)	13.4 (11.1–19.3)	13.2 (7.3–26.8)	0.601
	Neutrophils		11.1 (8.8–18.8)	11.1 (9.0–17.6)	12.2 (6.1–24.2)	0.482
	Lymphocytes		1.1 (0.6–1.5)	1.3 (0.6–2.2)	0.9 (0.4–1.4)	0.152
	Platelets		223.2 (129.1)	253.8 (111.1)	190.4 (142.6)	0.191
	NLR		12.6 (6.0–28.6)	7.0 (4.6–24.8)	14.4 (9.2–37.5)	0.269
	PLR		222.1 (125.4.-321.5)	235.2 (107.8–365.6)	207.7 (136.9–288.6)	0.568
	CRP		19.8 (11.3)	16.0 (10.3)	24.1 (11.0)	0.080
	PCT (ng/mL)	<0.5	7	2	5	-
		0.5–2	2	1	1	-
		2.1–10	8	6	2	-
		>10	4	1	3	-
	PCT		2.0 (0–2.0)	2.0 (0.8–2.0)	1.0 (0–3.0)	0.534
	Lactic acid		1.5 (0.95–2.32)	1.7 (1.0–3.4)	1.3 (0.9–2.2)	0.290
Comorbidities	CCI		7.0 (2.6)	7.2 (2.3)	6.7 (2.9)	0.620
	SOFA		9.7 (5.1)	8.7 (3.2)	10.7 (6.5)	0.286
	SAPS		48.3 (19.4)	46.1 (17.1)	50.6 (21.9)	0.550
Infection site	Urinary		12/29	4/15	8/14	0.099
	Pulmonary		9/29	6/15	3/14	0.250
	Abdominal		3/29	2/15	1/14	0.527
	Bacteremia		1/29	0/15	1/14	0.483
	Cutaneous		10/29	4/15	6/14	0.300
	Septic shock		18/29	11/15	7/14	0.181
Empirical therapy			19/29	10/15	9/14	0.600
Number of therapy days			6.5 (3.0)	5.8 (2.8)	7.2 (3.1)	0.198
30-day survival	No		12	5	7	-
	Yes		17	10	7	-

BMI: body mass index; CRP: C-reactive protein; CCI: Charlson Comorbidity Index; NLR: neutrophile–leucocyte ratio; PCT: procalcitonin (ng/mL), <0.5: in the reference range; 0.5–2, 2.1–10, >10: above reference range; PLR: platelet–leucocyte ratio; SAPS: Simplified Acute Physiology Score II; SOFA: Sequential Organ Failure Assessment Score; * statistically significant *p*-value ≤ 0.05.

**Table 2:** Pharmacokinetic parameters obtained in each group.

Variables	Treatment							
	II Group				CI Group			
	Mean	SD	CV%	Median	Mean	SD	CV%	Median
AUC0–24 (µg/mL*h)	159.12	150.44	94.54	111.91	116.50	61.44	52.74	109.29
Alpha (1/h)	1.74	2.44	140.66	1.21	1.40	1.84	131.32	0.56
Alpha_HL (h)	1.38	1.54	111.25	0.57	2.15	2.42	112.58	1.47
Beta (1/h)	0.09	0.08	93.00	0.07	0.10	0.07	62.68	0.11
Beta_HL (h)	20.87	21.56	103.27	9.25	11.55	10.74	92.96	6.20
CL (L/h)	7.88	7.02	89.13	5.36	6.95	4.11	59.16	5.66
CLD2 (L/h)	29.47	34.85	118.27	18.16	15.43	17.65	114.41	10.01
Cmax (µg/mL)	10.91	3.82	35.04	11.35	13.58	5.47	40.27	13.49
K10 (1/h)	0.18	0.16	90.19	0.10	0.22	0.12	53.83	0.23
K12 (1/h)	0.89	1.36	152.78	0.29	0.77	1.24	162.01	0.16
K21 (1/h)	0.75	1.11	148.01	0.44	0.52	0.61	118.76	0.22
V1 (L)	47.46	25.34	53.40	40.99	36.99	17.19	46.47	35.91
V2 (L)	26.50	22.25	83.95	24.01	26.17	26.37	100.74	23.75
Vss (L)	73.96	33.25	44.95	68.64	63.16	32.12	50.85	52.31

AUC0–24: area under the curve from 0 to 24 h; Alpha and Beta: distribution macro-constants; Alpha_HL: half-life of the distribution phase; Beta_HL: half-life of linezolid; CL: clearance; CLD2: distribution clearance; Cmax: maximum serum concentration; K10: elimination constant; K12: distribution constant from central (1) to peripheric (2) compartment; K21: distribution constant from peripheric (2) to central (1) compartment; h: hour; V1: distribution volume of central compartment; V2: distribution volume of peripheric compartment; Vss: distribution volume at steady state; SD: standard deviation; CV%: coefficient of variation (expressed as percentage).

**Table 3:** Pharmacokinetic parameters and PK/PD indices for each group.

PK Parameters	MIC (µg/mL)	II Group	SD	CI Group	SD	*p*
Mean serum concentration		9.57	7.00	8.79	4.09	0.717
Cmax (µg/mL)		10.91	3.82	13.58	5.47	0.138
Cmin (µg/mL)		6.63	6.31	-	-	-
Css (µg/mL)		-	-	9.01	4.16	-
Cmax/MIC (µg/mL)	2	5.46	1.91	6.79	2.73	0.137
	4	2.73	0.96	3.39	1.37	0.138
AUC_0–24_ (µg/mL*h)		159.12	150.44	116.50	61.44	0.333
AUC_24–48_ (µg/mL*h)		224.87	155.40	205.23	96.85	0.689
AUC_48–72_ (µg/mL*h)		236.40	182.50	222.04	106.09	0.801
AUC_24–48_/MIC ≥ 80 (%)	1	87	-	100	-	-
	2	60	-	64	-	-
	4	27	-	21	-	-
T > MIC > 80	1	14 out of 15 pts (93%)	-	14 out of 14 pts (100%)	-	-
	2	11 out of 15 pts (73%)	-	14 out of 14 pts (100%)	-	-
	4	10 out of 15 pts (67%)	-	13 out 14 pts (93%)	-	-

AUC0–24/24–48/48–72: area under de serum concentration–time curve in the 0–24 h/24–48 h/48–72 h periods; Cmax: maximum serum concentration; Cmin: trough serum concentration; Css: steady-state serum concentration; CL: drug clearance; MIC: minimum inhibitory concentration; PK: pharmacokinetics; PD: pharmacodynamics; SD: standard deviation; T > MIC > 80%: the percentage of time that the serum concentrations surpass the MIC. Statistically significant *p*-value ≤ 0.05.

**Table 4:** The influence of covariates on PK parameters.

	Hypothesis							
	Treatment	Sex	Age	Hepatic Function	Renal Function	Albumin	HD	BMI
Parameter (Unit)	*p*-Value							
AUC (h* µg mL)	0.167	0.419	0.260	0.111	0.244	0.972	0.224	0.722
Alpha (1/h)	0.621	0.240	0.236	0.354	0.695	0.241	0.602	0.363
Alpha_HL (h)	0.621	0.240	0.236	0.354	0.695	0.241	0.602	0.363
Beta (1/h)	0.090	0.773	0.341	0.082	0.239	0.968	0.120	0.991
Beta_HL (h)	0.090	0.773	0.341	0.082	0.239	0.968	0.120	0.991
CL (L/h)	0.167	0.419	0.260	0.111	0.244	0.972	0.224	0.722
CLD2 (L/h)	0.387	0.018 *	*0.084*	0.373	0.512	0.534	0.504	0.505
Cmax (µg/mL)	0.275	0.024 *	0.638	0.720	0.779	0.373	0.734	0.282
K10 (1/h)	0.901	0.618	0.192	0.054	0.095	0.970	0.159	0.801
K12 (1/h)	0.493	0.025 *	0.097	0.429	0.593	0.535	0.544	0.432
K21 (1/h)	0.980	0.607	0.125	0.136	0.474	0.458	0.201	0.456
V1 (L)	0.191	0.604	0.921	0.536	0.835	0.806	0.742	0.098
V2 (L)	0.384	0.023*	0.207	0.184	0.402	0.711	0.294	0.692
Vss (L)	0.078	0.233	0.567	0.280	0.491	0.817	0.295	0.899

AUC: area under serum concentration-time curve; Alpha: distribution macro-constant; Alpha_HL: half-life of alpha (distribution) phase; Beta: distribution macro-constant; Beta_HL: half-life of beta (distribution) phase; CL: drug clearance; CLD2: distribution clearance; Cmax: maximum serum concentration; K10: elimination constant; K12: distribution constant from compartment 1 (central compartment) to 2 (peripheric compartment); K21: distribution constant from compartment 2 (peripheric compartment) to 1 (central compartment); V1: distribution volume of central compartment; V2: distribution volume of peripheric compartment; Vss: distribution volume in steady state. * Statistically significant *p*-value ≤ 0.05 (ANOVA).

## Data Availability

The original contributions presented in the study are included in the article; further inquiries can be directed to the corresponding author.

## Supplementary Materials

The following supporting information can be downloaded at: https://www.mdpi.com/article/10.3390/antibiotics13100961/s1, Table S1: Past published studies that analyzed continuous infusion of linezolid.
