# Supplementary material for: Linezolid Pharmacokinetics in Critically Ill Patients: Continuous Versus Intermittent Infusion
Source: Antibiotics (Basel). 2024 Oct 11;13(10):961. doi: 10.3390/antibiotics13100961 (PMC11504488; doi:10.3390/antibiotics13100961)
Supplement: Supplementary file 1 [file antibiotics-13-00961-s001.zip › antibiotics-3205349-supplementary.pdf]

Supplementary data

Table S1. Past published studies that analyzed continuous infusion of linezolid.

No.	Reference, year of publication	Study type	Randomization	Antibiotic group			Case definition
				**CI group**	**II group**	**Simulation**	
1	Wicha et al, 2022[15]	Retrospective observational study	No	120 pts	ND	MCs of various dosing from 300mg to 2400 mg	Critically ill pts with sepsis/septic shock
**2**	**Warda et al, 2022[16]**	**Prospective controlled trial, one center, two arms**	**Yes**	**92**	**87**	**ND**	***Critically ill* patients with G+ nosocomial pneumonia**
**3**	**Barrasa et al, 2020[17]**	**Prospective, open-label, multi-center, two arm+MCs**	**No**	**26 pts with 1200mg CI/day**	**17 pts with 30 min iv of 600mg q 12h**	**MCs on 1000 subjects**	***Critically ill***
4	Soraluce et al, 2020[18]	Prospective, open-label, multi-center, two arm	No	11 pts with 1200mg CI/day	40 pts with 30 min iv of 600mg q12h		*Critically ill*
5	Bohle et al, 2020[19]	Prospective, observational, single center, single arm	No	25 pts with 600mg iv loading dose + 1200mg CI/day	ND		ICU pts
6	Kuhn et al, 2020[20]	Prospective, observational, single center, single arm	No	19 pts with 600mg iv loading dose+1800mg as CI/day	ND		ICU pts with severe respiratory and bloodstreem infection
7	Barrasa et al, 2017[21]	Prospective, multi-center, single arm. Poster	No	22 pts wih 600mg iv loading dose+1200mg as CI/day	ND		*Critically ill*
8	Protti et al, 2016[22]	Case report	No	1 pt with 1200mg CI/day	ND	ND	Post transplant pneumonia pts.
9	Alvarez-Lerma et al, 2016[23]	Case report	No	1 pt with 1800mg CI/day	1 pt with 60 min iv of 600mg q 12h	ND	Septic shock secondary to community acquired MRSA pneumonia
10	De Pascale et al, 2015[24]	Prospective, controlled, two arm + MCs on 1000 situations	Yes	11 pts with 600mg iv loading dose+1200mg as CI/day	11 pts with 60 min iv of 600mg q 12h	MCs on 1000 situations	*Critically ill* obese pts with VAP.
11	De Pascale et al, 2013[25]	Prospective, single center, two arm. Abstracts volume	Yes	7 pts with 600mg iv loading dose+1200mg as CI/day	7 pts with 600mg iv q 12h		*Critically ill* obese pts with nosocomial pneumonia due to suspected MRSA
12	Boselli et al, 2012[26]	Prospective, open-label, single center, single arm	No	12 pts with 1 h iv of 600mg, followed by 1200mg as CI/day	ND	ND	*Critically ill* adult pts with VAP
13	Tascini et al, 2011[27]	Research letter	No	2 pts with 600mg iv q 12h and who continued with 1200mg as CI/day.	8 pts with 600mg iv q 12h	ND	Endocarditis in patients with native or prosthetic valve or pacemaker
14	Adembri et al, 2008[28]	Prospective, open-lable, single center, two arm	Yes	8 pts with 30 min iv of 300mg+900mg as CI in day 1, followed by 1200mg as CI	8 pts with 30 min iv of 600mg q12h	ND	Septic *critically ill* ICU adult patients

Pt(s)—patient(s), IV—intravenous, CI—continuous infusion, II—intermittent infusion, CrCl—creatinine clearance, q—every, VAP—ventilation associated pneumonia, min—minutes, ND—not determined, ICU—intensive care unit, G+—gram positive bacteria, MRSA—methicillin-resistant *Staphylococcus aureus*, MCs—Monte Carlo simulation, ARDS—acute respiratory distress syndrome, C—concentration, PK—pharmacokinetic. Comparative studies that used more that 15 patients in the CI group —in **bold.**
